# Computed high-*b*-value high-resolution DWI improves solid lesion detection in IPMN of the pancreas

**DOI:** 10.1007/s00330-023-09661-6

**Published:** 2023-05-03

**Authors:** Felix N. Harder, Eva Jung, Kilian Weiss, Markus M. Graf, Omar Kamal, Sean McTavish, Anh T. Van, Ihsan E. Demir, Helmut Friess, Veit Phillip, Roland M. Schmid, Fabian K. Lohöfer, Georgios A. Kaissis, Marcus R. Makowski, Dimitrios C. Karampinos, Rickmer F. Braren

**Affiliations:** 1 Institute of Diagnostic and Interventional Radiology, Technical University of Munich, School of Medicine, Munich, Germany; 2 Philips GmbH Market DACH, Röntgenstrasse 22, 22335 Hamburg, Germany; 3 Department of Surgery, Klinikum Rechts Der Isar, School of Medicine, Technical University of Munich, Munich, Germany; 4 Department of Medicine II, University Hospital Rechts Der Isar, Technical University Munich, Munich, Germany; 5 Department of Computing, Faculty of Engineering, Imperial College of Science, Technology and Medicine, London, SW7 2AZ UK; 6 Institute for Artificial Intelligence in Medicine, Technical University of Munich, Munich, Germany; 7 Munich Institute of Biomedical Engineering, Technical University of Munich, Garching, Germany

**Keywords:** Diffusion magnetic resonance imaging, Pancreas, Pancreatic neoplasms, Pancreatic cysts

## Abstract

**Objectives:**

To examine the effect of high-*b*-value computed diffusion-weighted imaging (cDWI) on solid lesion detection and classification in pancreatic intraductal papillary mucinous neoplasm (IPMN), using endoscopic ultrasound (EUS) and histopathology as a standard of reference.

**Methods:**

Eighty-two patients with known or suspected IPMN were retrospectively enrolled. Computed high-*b*-value images at *b* = 1000 s/mm^2^ were calculated from standard (*b* = 0, 50, 300, and 600 s/mm^2^) DWI images for conventional full field-of-view (fFOV, 3 × 3 × 4 mm^3^ voxel size) DWI. A subset of 39 patients received additional high-resolution reduced-field-of-view (rFOV, 2.5 × 2.5 × 3 mm^3^ voxel size) DWI. In this cohort, rFOV cDWI was compared against fFOV cDWI additionally. Two experienced radiologists evaluated (Likert scale 1–4) image quality (overall image quality, lesion detection and delineation, fluid suppression within the lesion). In addition, quantitative image parameters (apparent signal-to-noise ratio (aSNR), apparent contrast-to-noise ratio (aCNR), contrast ratio (CR)) were assessed. Diagnostic confidence regarding the presence/absence of diffusion**-**restricted solid nodules was assessed in an additional reader study.

**Results:**

High-*b*-value cDWI at *b* = 1000 s/mm^2^ outperformed acquired DWI at *b* = 600 s/mm^2^ regarding lesion detection, fluid suppression, aCNR, CR, and lesion classification (*p* =  < .001–.002). Comparing cDWI from fFOV and rFOV revealed higher image quality in high**-**resolution rFOV-DWI compared to conventional fFOV-DWI (*p* ≤ .001–.018). High-*b*-value cDWI images were rated non-inferior to directly acquired high-*b*-value DWI images (*p* = .095–.655).

**Conclusions:**

High-*b*-value cDWI may improve the detection and classification of solid lesions in IPMN. Combining high-resolution imaging and high-*b*-value cDWI may further increase diagnostic precision.

**Clinical relevance statement:**

This study shows the potential of computed high-resolution high-sensitivity diffusion-weighted magnetic resonance imaging for solid lesion detection in pancreatic intraductal papillary mucinous neoplasia (IPMN). The technique may enable early cancer detection in patients under surveillance.

**Key Points:**

• *Computed high-b-value diffusion-weighted imaging (cDWI) may improve the detection and classification of intraductal papillary mucinous neoplasms (IPMN) of the pancreas.*

• *cDWI calculated from high-resolution imaging increases diagnostic precision compared to cDWI calculated from conventional-resolution imaging.*

• *cDWI has the potential to strengthen the role of MRI for screening and surveillance of IPMN, particularly in view of the rising incidence of IPMNs combined with now more conservative therapeutic approaches.*

**Supplementary Information:**

The online version contains supplementary material available at 10.1007/s00330-023-09661-6.

## Introduction

Cross-sectional imaging has led to an increased discovery rate of pancreatic intraductal papillary mucinous neoplasms (IPMN) [1]. IPMN represent precancerous lesions developing from the mucinous epithelium of the pancreatic duct [2]. IPMN management ranges from surveillance to surgical resection, depending on the risk of a malignant transformation, which is estimated from imaging features, clinical symptoms, and laboratory values [3]. Magnetic resonance imaging (MRI) has become a central diagnostic pillar in major international guidelines on the management of IPMN [3-5].

Whereas T2-weighted (T2w) imaging and magnetic resonance cholangiopancreaticography (MRCP) are highly sensitive for the detection and quantification of the fluid component, diffusion-weighted imaging (DWI) is highly sensitive for the detection of the solid component, which indicates malignant transformation [6, 7]. Previous studies showed promising results for high-*b*-value DWI in the identification of solid components in IPMN using *b* values up to 800 s/mm^2^ [8]. Similarly, *b* values of b ≥ 1000 s/mm^2^ improve the detection rate in pancreatic ductal adenocarcinoma [9]. However, the application of increased *b* values comes with longer acquisition times and exposition to motion artifacts [10].

Computed high-*b*-value DWI (cDWI) from at least two acquired lower *b* values based on a voxel-wise mono-exponential fit has been proposed to overcome the above-mentioned limitations [11-14]. Moreover, high-resolution DWI has been reported to increase image quality for both pancreatic DWI and cDWI [15, 16]. As diagnostic reliability in DWI stems from the signal intensity and image quality, integrating both cDWI and high-resolution DWI might strengthen the role of DWI in IPMNs.

Our primary aim was to compare the value of cDWI at *b* = 1000 s/mm^2^ against acquired DWI at *b* = 600 s/mm^2^ in IPMN.

We further investigated the value of high-resolution cDWI in pancreatic IPMN with regard to qualitative and quantitative parameters as well as diagnostic accuracy. Endoscopic ultrasound (EUS) and histopathology served as the standard of reference.

## Material and methods

### Study design

This study was designed as a single-center observational study. All patients undergoing the additional high-resolution DWI sequence were prospectively enrolled, and informed consent was given (IRB Protocol Nr. 102/21 S-EB). All other patients were retrospectively enrolled, and the requirement for informed consent was waived (IRB Protocol Nr. 180/17S). The study was conducted in accordance with the Declaration of Helsinki.

All patients were referred to the departments of surgery or internal medicine for work-up of suspected IPMN or follow-up of known IPMN. Patients who underwent an in-house MRI between February 2018 and June 2021 were included. Exclusion criteria were (1) IPMN size < 10 mm, (2) non-diagnostic image quality, (3) incomplete MRI datasets, and (4) entity other than IPMN. The patient inclusion flowchart can be found in the supplementary material (S.1).

The following clinical data were obtained: age at diagnosis, sex, IPMN type (main duct, side branch, mixed type) based on imaging criteria, and EUS as well as histopathology when available. EUS was performed by an experienced (> 7 years) gastroenterologist with specialization in EUS (Table 1).

**Table 1:** Characteristics of all patients included for quantitative and qualitative image analyses

Variable	*n* = 82
Age	
Mean (years)	72
Range	43–89
Stdev	10.14
Sex	
Male	51 (62%)
Female	31 (38%)
Lesion	
BD-IPMN	67 (81.7%)
MD-IPMN	7 (8.5%)
MT-IPMN	8 (9.8%)
EUS	77 (93.9%)

### Data acquisition and postprocessing

MRI datasets were acquired on a whole-body 3-T MRI system (Philips Ingenia Elition; Philips Medical Systems) using a combination of a 16-channel torso coil array and an inbuilt table posterior 12-channel coil array.

**Fig. 1:**
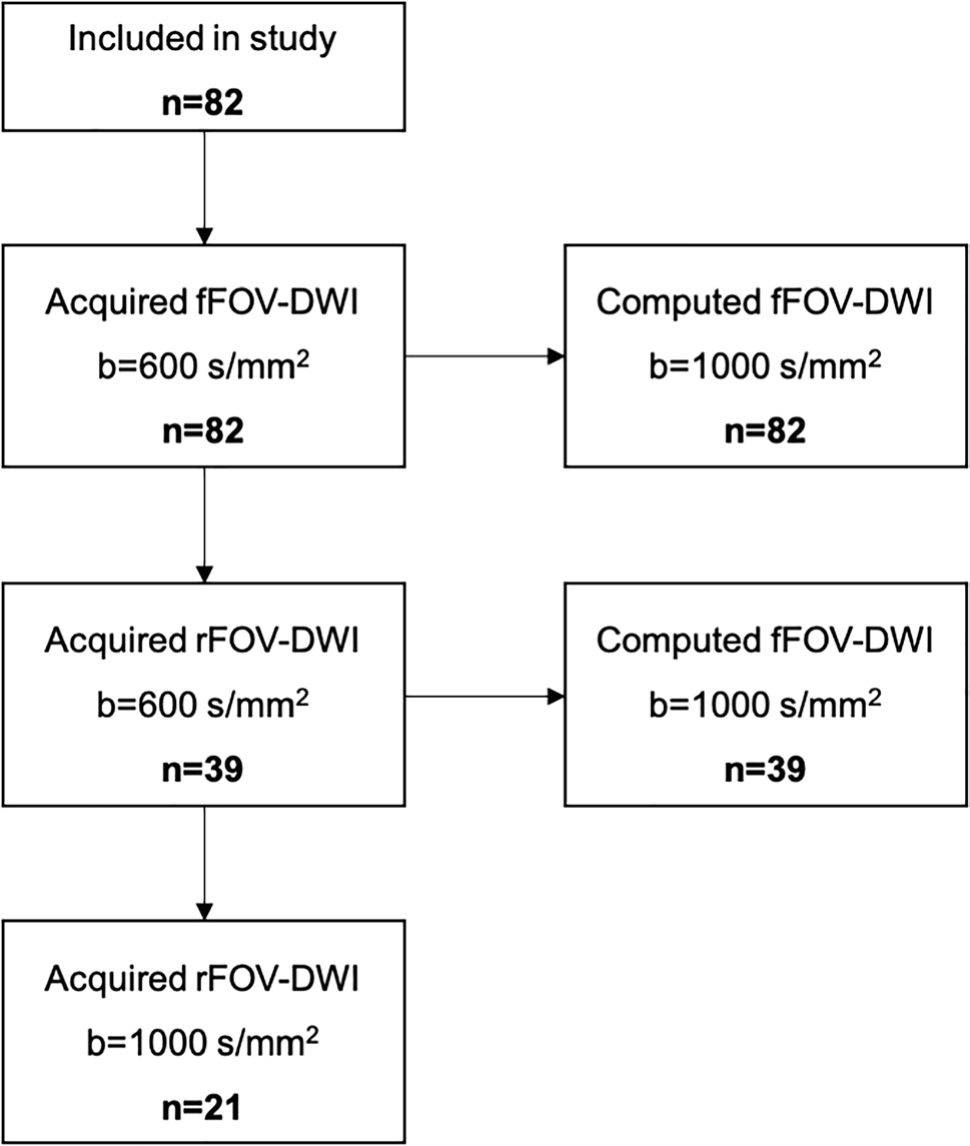
Image acquisition flow chart

A T2-weighted (T2w) turbo spin echo (TSE) sequence and a conventional (i.e., full field of view, fFOV) 2D diffusion-weighted (DW) single-shot echo-planar imaging (ssEPI) sequence for whole upper abdomen coverage were acquired in all 88 patients. fFOV images were acquired at the following *b* values (averages) as routinely performed at our institution: 0 (1), 50 (1), 300 (2), 600 (5) s/mm^2^. In a subset of 39 patients, a high-resolution (i.e., reduced field of view, rFOV) 2D DW single-shot echo EPI sequence, covering the pancreas, was performed additionally, at the same *b* values as the fFOV sequence. Furthermore, in a subset of 21 patients with high-resolution DWI, an additional high *b* value at *b* = 1000 (11) s/mm^2^ was obtained (r-aDWI1000). Figure 1 displays the image acquisition flow chart. Datasets were acquired in axial planes with respiratory triggering. Further sequence parameters are displayed in Table 2.

**Table 2:** Sequence parameters

Acquisition parameters		
	fFOV-DWI	rFOV-DWI
TE/TR (ms)	72/1850	67/1627
FOV (mm^2^)	420 × 370	300 × 300
Voxel size (mm^3^)	3 × 3 × 4	2.5 × 2.5 × 3
Slices	43	20
Bandwith (Hz/pixel)	2304	2817
Parallel imaging factor (SENSE)	2.5	2.5
Phase encoding	A/P	L/R
*b* values (averages) (s/mm^2^)	0 (1), 50 (1), 300 (2), 600 (5)-	0 (1), 50 (1), 300 (2), 600 (5), and 1000 (11)
Scan time (min)	4:30	3:18 min (b0-600 s/mm^2^) 15:00 min (b0-1000 s/mm^2^)

A dedicated software tool (Philips IntelliSpace Portal, Philips Medical Systems) was utilized to generate cDWI images. Therefore, *b* values of 0, 50, 300, and 600 s/mm^2^ were used to calculate cDWI images at a *b* value of 1000 s/mm^2^, based on a mono-exponential fit model, for both fFOV (f-cDWI1000) and rFOV (r-cDWI1000).

### Image analysis

To assess performances, conventional (i.e., fFOV) and high-resolution (i.e., rFOV) calculated high-*b*-value cDWI (f-cDWI1000, r-cDWI1000) were compared (i) to the corresponding acquired *b* = 600 s/mm^2^ images (f-aDWI600, r-aDWI600), (ii) to each other, and (iii) against acquired high-resolution high-*b*-value images (r-aDWI1000).

### Qualitative analysis

Two experienced radiologists with 4 (reader 1) and 10 (reader 2) years of experience performed qualitative image analysis blinded to each other’s results. In all 88 patients, the following qualitative image parameters were rated on a 4-point Likert scale: overall image quality (4 = excellent, 3 = good, 2 = fair, 1 = poor), lesion detection and delineation (4 = excellent, 3 = good, 2 = fair, 1 = poor), and fluid suppression within the lesion (4 = excellent, 3 = good, 2 = fair, 1 = poor).

### Quantitative analysis

A 5 mm measuring region of interest (ROI) was manually placed in the IPMN and the healthy-appearing tissue next to the lesion in the pancreatic head and tail. Nodules and prominent septations within the IPMN were omitted if possible. ROIs were placed in the *b* = 600 s/mm^2^ images and then copied and pasted to the remaining images. The mean signal intensity (SI) and standard deviation (SD) inside the ROIs were recorded.

The apparent signal-to-noise ratio (aSNR) was calculated as follows:

$$\mathrm{aSNR}={\mathrm{SI}}_{\mathrm{normal\; parenchyma}}/{\mathrm{SD}}_{\mathrm{normal \;parenchyma}}.$$

The apparent contrast-to-noise ratio (aCNR) was calculated as follows:

$$\mathrm{aCNR}=\left({\mathrm{SI}}_{\mathrm{tumor}}-{\mathrm{SI}}_{\mathrm{normal\; parenchyma}}\right)/{\mathrm{SD}}_{\mathrm{normal\; parenchyma}}.$$

The contrast ratio between the lesion and the adjacent normal parenchyma was calculated as follows:

$$\mathrm{CR}=\mathrm{\;SI} \mathrm{\;lesion}/\mathrm{SI}\; \mathrm{normal \;pancreas}.$$

### Reader study

We implemented a prospectively designed reader study to investigate whether alterations in qualitative and quantitative imaging parameters would affect radiological decision-making. Both readers were asked to qualitatively rate their diagnostic confidence regarding the presence or absence of diffusion-restricted nodules on a 4-point Likert scale (4 = definitely nodule, 3 = probably nodule, 2 = probably no nodule, 1 = no nodule) for acquired DWI and cDWI images. Both readers were blinded to the EUS result. A score of 3 or 4 implicated further work-up by EUS. A score of 1 or 2 would implicate no further EUS examination.

To provide consistency between the MRI and EUS findings, we searched our cohort for patients who underwent EUS for suspected IPMN after MRI or no longer than 3 months prior to the MRI. All eligible patients were included in the prospectively designed reader study. A total of 62 out of 88 patients in our cohort met the criteria for inclusion. In 5 patients, EUS and MRI were performed on the same day. In 24 patients, EUS was performed prior to the MRI scan (median: 25 days, IQR: 33 days), and in 33 patients, the EUS was performed after the MRI scan (median: 56 days, IQR: 191).

Furthermore, three patients without EUS but with stable follow-up MRI as well as one patient with a stable follow-up PET-CT over the course of 2 years were included. One additional patient without EUS, but for whom pancreatectomy was performed within 3 months after the MRI, was also included. Finally, 67 patients were enrolled in the reader study.

In 18/67 (27%) patients, EUS revealed a solid nodule within the IPMN, indicating potential malignant transformation. Based on imaging findings and clinical indication, 20/67 patients were resected and histopathological correlation revealed malignancy in 13/20 patients. Histopathological examination was performed by experienced pathologists with a specialization in pancreatic pathologies.

### Statistical analysis

Qualitative and quantitative metrics were analyzed using the Wilcoxon signed-rank test. Receiver operating curve (ROC) analysis was applied for diagnostic confidence. Sensitivity and specificity calculations were performed by binarising the Likert scale so that scores 1 and 2 were considered “negative” and 3 and 4, “positive.” ROC curves were calculated by assigning a probability of 0.25 to Likert scale 1, 0.50 to 2, 0.75 to 3, and 1.0 to 4. The DeLong test for paired samples was used to compare areas under the ROC curves.

Inter-rater agreement was calculated using Cohen’s *κ*. Agreement was considered as *slight*: *κ* = 0.00–0.20; *fair*: *κ* = 0.21–0.40, *moderate*: *κ* = 0.41–0.60, *substantial*: *κ* = 0.61–0.80, and *almost perfect*: *κ* = 0.81–1.00. *p* values ≤ 0.05 were considered statistically significant. All statistics were performed in *IBM SPSS* (version 28) and Python, version 3.9.2.

## Results

### Patient characteristics

A total of 82 patients were finally enrolled. Patient characteristics can be found in Table 1.

### Qualitative parameters

#### Image quality

Image quality was significantly higher in *b* = 600 s/mm^2^ images compared to cDWI images, holding true for both conventional and high-resolution DWI (f-aDWI600 3.33 ± 0.56, f-cDWI1000 2.58 ± 0.72, *p* < 0.001; r-aDWI600 3.60 ± 0.58, r-cDWI1000 3.23 ± 0.61, *p* = 0.0019) (Tables 3 and 4). Comparing cDWI images revealed significantly higher image quality in high-resolution cDWI (f-cDWI1000 2.61 ± 0.72, r-cDWI1000 3.23 ± 0.61, *p* < 0.001). No statistically significant difference was found in directly acquired compared to computed high-resolution images (r-cDWI1000 3.23 ± 0.61, r-aDWI1000 3.55 ± 0.74, *p* = 0.095). High inter-rater agreement of 0.79–0.92 was found.

**Table 3:** Ratings for the assessed qualitative image parameters

Category	f-aDWI600	f-cDWI1000	r-aDWI600	r-cDWI1000	r-aDWI1000
Image quality	3.3 ± .56	2.58 ± .72	3.60 ± .58	3.23 ± .61	3.55 ± .74
Lesion detection	2.67 ± 1.2	3.48 ± .61	3.79 ± .41	3.73 ± .46	3.73 ± .46
Fluid suppression	1.22 ± .5	3.05 ± .7	1.70 ± 1	3.77 ± .43	3.55 ± .74
Diagnostic confidence	2.10 ± .7	1.69 ± .99	2.09 ± .84	1.53 ± 1.1	1.55 ± 1.1

**Table 4:** Corresponding *p* values for comparison of the qualitative image parameters

Category	f-aDWI_600_ vs f-cDWI_1000_	r-aDWI_600_ vs r-cDWI_1000_	f-cDWI_1000_ vs r-cDWI_1000_	r-aDWI_1000_ vs r-cDWI_1000_
Image quality	< .001	.0019	< .001	0.095
Lesion detection	< .001	< .0019	< .001	.42
Fluid suppression	< .001	< .001	< .001	.24
Diagnostic confidence	< .001	.002	.018	.655

#### Lesion detection and delineation

**Fig. 2:**
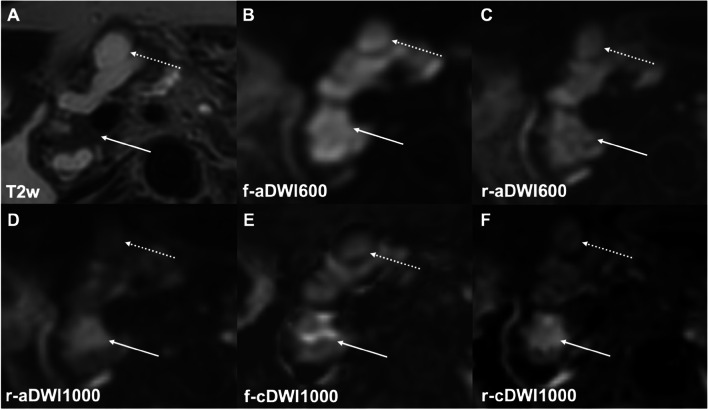
A mixed-type IPMN in a 75-year-old female patient. The solid tissue component in the pancreatic head (T2w, arrow) is not visible in both conventional and high-resolution DWI at *b* = 600 s/mm^2^ (**B**, **C**) and led to the false diagnosis of a benign IPMN. Also, the conventional high-*b*-value computed image indicates no clear diffusion restriction (**D**). However, the high-resolution high-*b*-value computed image (**E**) led to the correct diagnosis of malignant IPMN, which was confirmed pathologically after resection. Fluid suppression (dotted arrows) is best achieved in r-cDWI1000. Furthermore, high-resolution high-*b*-value computed DWI outperforms directly acquired high-resolution high-*b*-value DWI (**F**) regarding image quality and fluid suppression

**Fig. 3:**
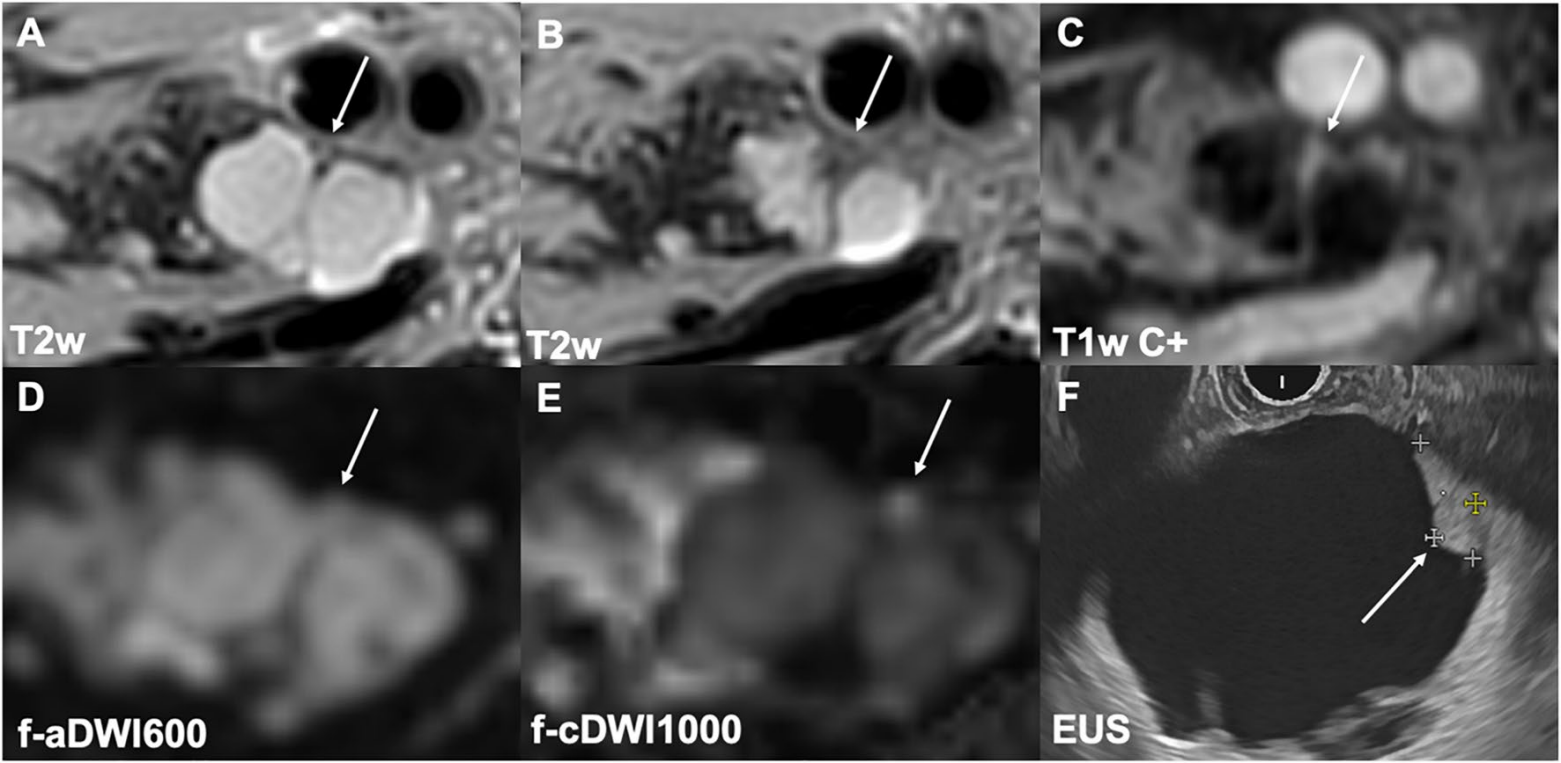
In an 81-year-old female patient, T2w (**A**, **B**) and contrast-enhanced T1w (**C**) images depict a BD-IPMN in the pancreatic head exhibiting focal contrast enhancement (arrow in **C**). The mural nodule is clearly better detectable in the high-*b*-value computed DWI (arrow in **E**) compared to the standard *b*-value DWI (arrow in **D**). Endoscopic ultrasound (**F**) confirms the presence of a suspicious enhancing mural nodule (arrow). Histopathological analysis after resection confirmed an IPMN with concomitant cancer

cDWI images outperformed the corresponding *b* = 600 s/mm^2^ images regarding lesion detection and delineation (f-aDWI600 2.67 ± 1.2, f-cDWI1000 3.48 ± 0.61, *p* < 0.001; r-aDWI600 3.76 ± 0.48, r-cDWI1000 3.79 ± 0.41, *p* = 0.0019) (Tables 3 and 4; Figs. 2 and 3). In the subgroup of 39 patients receiving conventional and high-resolution imaging, high-resolution cDWI images were rated superior compared to conventional cDWI images (r-cDWI1000 3.79 ± 0.41, f-cDWI1000 3.49 ± 0.63, *p* < 0.001) (Fig. 2). No difference was found between computed and directly acquired high-resolution images (r-cDWI1000 3.79 ± 0.41, r-aDWI1000 3.73 ± 0.46, *p* = 0.42). Inter-rater agreement was high, with values between 0.74 and 0.95*.*

### Fluid suppression

**Fig. 4:**
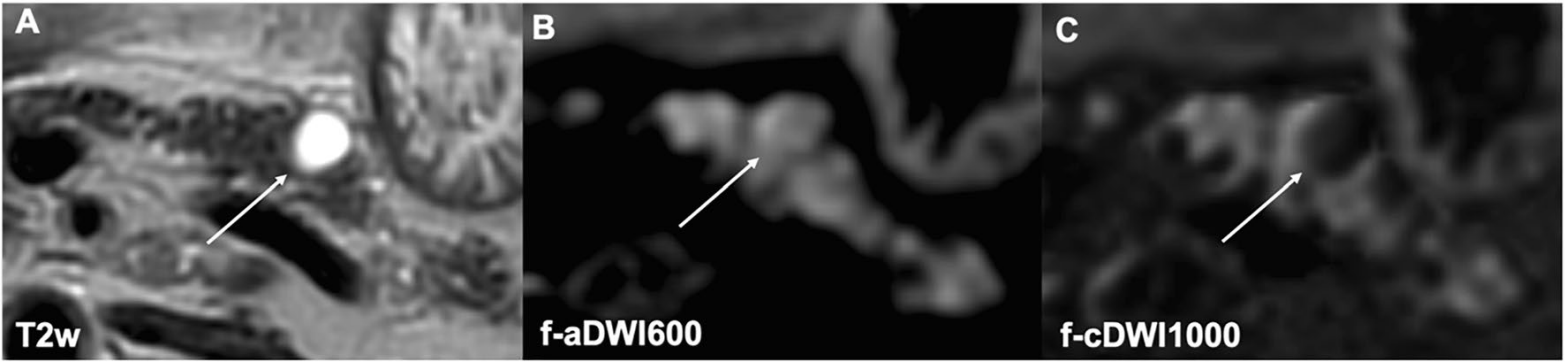
A branch-duct IPMN in the pancreatic body is depicted in the axial T2w image (**A**). Better fluid suppression within the lesion is seen in the high-*b*-value computed image (**C**) compared to the standard *b*-value DWI (**B**)

**Fig. 5:**
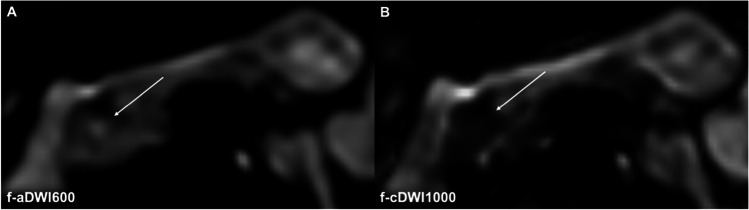
73-year-old women with a benign IPMN. Malignant IPMN was misdiagnosed based on the standard DWI at *b* = 600 s/mm^2^ (**A**) but correctly diagnosed as benign reviewing the computed high-*b*-value DWI at *b* = 1000 s/mm.^2^ (**B**)

Fluid suppression within the IPMN was rated significantly higher in cDWI images compared with the corresponding acquired *b* = 600 s/mm^2^ images (f-aDWI600 1.22 ± 0.5, f-cDWI1000 3.05 ± 0.7, *p* < 0.001; r-aDWI600 1.70 ± 1, r-cDWI1000 3.77 ± 0.43, *p* < 0.001) (Tables 3 and 4, Figs. 4 and 5). Furthermore, high-resolution cDWI images outperformed conventional cDWI images (cDWI1000 3.77 ± 0.43, f-cDWI1000 3.06 ± 0.7, *p* < 0.001). No significant difference was seen in computed compared to directly acquired high-resolution high-*b*-value images (r-cDWI1000 3.77 ± 0.43, r-aDWI1000 3.55 ± 0.74, *p* = 0.24). Again, Cohen’s *κ* revealed high inter-rater agreement with 0.8–0.96.

### Quantitative parameters

#### aSNR

Mean aSNR was significantly higher in *b* = 600 s/mm^2^ images compared to cDWI images (f-aDWI600 14.1 ± 3.9, f-cDWI1000 11.06 ± 0.5.78, *p* = 0.011; r-aDWI600 10.56 ± 2.27, r-cDWI1000 9.06 ± 1.18, *p* = 0.005) (Table 5).

**Table 5:** The corresponding false-positive rate (FPR), false-negative rate (FNR), true-positive rate (TPR), and true-negative rate (TNR) obtained from the reader study based on EUS as the gold standard

Category	f-aDWI600	f-cDWI1000	r-aDWI600	r-cDWI1000	r-aDWI1000
FPR	0.229	0.412	0.269	0.038	0.1
FNR	0.684	0.263	0.5	0.125	0.4
TPR	0.316	0.737	0.5	0.875	0.6
TNR	0.771	0.958	0.731	0.962	0.9

Higher mean aSNR was found in conventional cDWI images compared to high-resolution cDWI images, yet not reaching statistical significance (f-cDWI1000 11.06 ± 0.5.78, r-cDWI1000 9.06 ± 1.18, *p* = 0.17). Significantly higher aSNR was found in high-resolution cDWI images compared to directly acquired high-*b*-value images at *b* = 1000 s/mm^2^ (r-cDWI1000 9.06 ± 1.18, r-aDWI1000 8.42 ± 1.18, *p* = 0.005).

#### aCNR

Mean aCNR was significantly higher in both cDWI images compared to acquired images, holding true for both conventional and high-resolution DWI (f-aDWI600 2.91 ± 1.6, f-cDWI1000 4.37 ± 1.84, *p* = 0.023; r-aDWI600 3.31 ± 2.06, r-cDWI1000 5.35 ± 1.43, *p* = 0.008) (Table 5). Also, higher mean aSNR was found in high-resolution cDWI compared to conventional cDWI (r-cDWI1000 5.35 ± 1.43, f-cDWI1000 4.37 ± 1.84, *p* = 0.017) as well as compared to directly acquired high-resolution *b* = 1000 s/mm^2^ images (r-cDWI1000 5.35 ± 1.43, r-aDWI1000 3.7 ± 1.49, *p* = 0.001).

#### CR

CR between the IPMN and proximal, as well as distal, pancreatic parenchyma was significantly higher in high-resolution cDWI images compared to *b* = 600 s/mm^2^ images (proximal: f-aDWI600 1.22 ± 0.19, f-cDWI1000 2.13 ± 1.14, *p* < 0.001; r-aDWI600 1.35 ± 0.29, r-cDWI1000 3.21 ± 2.44, *p* < 0.001; distal: f-aDWI600 1.32 ± 0.29, f-cDWI1000 2.14 ± 1.46, *p* = 0.012; r-aDWI600 1.42 ± 0.24, r-cDWI1000 2.8 ± 1.54, *p* < 0.001) (Table 5).

Comparing conventional cDWI to high-resolution cDWI revealed significantly higher CR in high-resolution images (proximal: *p* = 0.013, distal: *p* = 0.035). Computed high-resolution cDWI outperformed respective acquired images at *b* = 1000 s/mm^2^ (proximal: r-cDWI1000 3.21 ± 2.44, r-aDWI1000 1.92 ± 0.57, *p* = 0.005; distal: r-cDWI1000 2.8 ± 1.54, r-aDWI1000 1.88 ± 0.62, *p* = 0.004).

### Reader study

Likert scale ratings differed significantly between the *b* = 600 s/mm^2^ images and the corresponding cDWI images in both conventional and high-resolution DWI (f-aDWI600 2.10 ± 0.7, f-cDWI1000 1.69 ± 0.99, *p* < 0.001; r-aDWI600 2.09 ± 0.84, r-cDWI1000 1.53 ± 1.1, *p* = 0.002). A significant difference was also found comparing both cDWI images (*p* = 0.018). No significant difference was found comparing high-resolution cDWI images versus directly acquired images at *b* = 1000 s/mm^2^ (r-cDWI1000 1.53 ± 1.1, r-aDWI1000 1.55 ± 1.1, *p* = 0.665). Inter-rater agreement was 0.77–0.94.

**Fig. 6:**
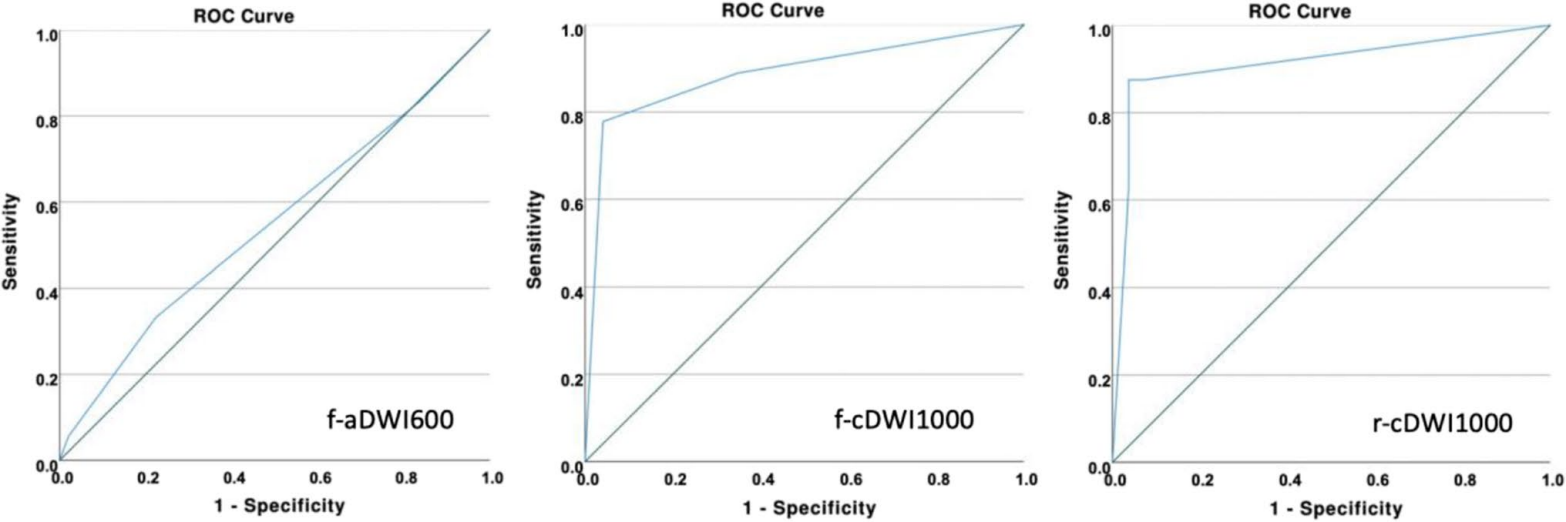
displays the ROC curves for f-aDWI600 (0.54), f-cDWI1000 (0.86), and r-cDWI1000 (0.91)

Conventional *b* = 600 s/mm^2^ images received a sensitivity and specificity of 0.32 and 0.77, respectively, whereas sensitivity and specificity were 0.73 and 0.96, respectively, in conventional cDWI images. Likewise, the ROC was significantly higher in cDWI (f-aDWI600 0.54, f-cDWI1000 0.86, *p* < 0.0001) (Fig. 6). The same finding was seen in high-resolution images. High-resolution *b* = 600 s/mm^2^ images received a sensitivity and specificity of 0.50 and 0.73, respectively, whereas sensitivity and specificity were 0.88 and 0.96, respectively, in high-resolution cDWI images. Again, the ROC was significantly higher in high-resolution cDWI (r-aDWI600 0.63, f-cDWI1000 0.91, *p* = 0.018) (Fig. 6).

Comparison between both cDWI images revealed a sensitivity, specificity, and ROC of 0.75, 0.93, and 0.83, respectively, for conventional images and 0.88, 0.96, and 0.91 for high-resolution images; *p* = 0.52.

Comparing cDWI versus acquired high-resolution images showed a sensitivity, specificity, and ROC of 0.6, 0.9, and 0.72, respectively, for acquired images and 0.9, 0.9, and 0.84, respectively, for cDWI images; *p* = 0.27.

## Discussion

In our study, we examined the diagnostic performance of high-*b*-value cDWI and the added value of high-resolution high-*b*-value cDWI in pancreatic IPMN. Calculated high-*b*-value cDWI outperformed acquired DWI at *b* = 600 s/mm^2^ regarding lesion detection and diagnostic precision. Further diagnostic accuracy was achieved by combining cDWI and high**-**resolution DWI. Overall, the best image quality and highest diagnostic precision were found in high-resolution high-*b*-value cDWI. Of note, high-resolution cDWI was comparable to directly acquired high-resolution high-*b*-value DWI.

The presence of mural nodules in imaging studies is a strong indicator of a malignant transformation of an IPMN [4, 5]. According to current European guidelines, the presence of a solid mass is considered an absolute indication for surgery in IPMN [4].

We found cDWI images to outperform DWI at *b* = 600 s/mm^2^ regarding lesion detection and delineation. In our sample, cDWI images revealed a significantly increased diagnostic accuracy in detecting solid nodules within IPMNs, reflected by higher sensitivity and specificity, compared to *b* = 600 s/mm^2^ images. This subjective finding was confirmed by the significantly higher CR and CNR between the IPMN and the adjacent healthy pancreatic parenchyma and is attributable to the stronger fluid suppression in cDWI images.

Incomplete fluid suppression at *b* = 600 s/mm^2^ not only obscures small diffusion-restricted nodules but also mimics solid components within IPMNs. Our results are in line with a previous study on cDWI in pancreatic ductal adenocarcinoma, reporting more accurate lesion detection *b* = 1000 s/mm^2^ [17].

Comparing conventional and high-resolution cDWI revealed increased lesion detection and higher ROC in favor of the high-resolution images. Improved lesion detection in rFOV-DWI compared to fFOV-DWI has been reported previously [16]. In a recent meta-analysis**,** Xu et al reported that DWI slice thickness affected the diagnostic performance in discriminating benign from malignant IPMN [18]. Hence, reduced T2 shine-through effect with higher *b* values and increased spatial resolution with thinner slices contribute to the improved diagnostic accuracy in high-*b*-value high-resolution DWI as found herein.

Our findings may be important in view of the paradigm shift in IPMN treatment, evolving from early surgical resection—with the risk of potential overtreatment—to close surveillance. Surveillance schemes vary among major guidelines, in part due to diverging results in favor of either MRI or EUS [19-21]. However, the major advantage of MRI is its non-invasive nature. Hence, advances in MRI could strengthen the role of MRI in IPMN management.

Image quality decreased in cDWI compared to *b* = 600 s/mm^2^ images in our study. Reduced image quality has been recently reported in cDWI of the pancreas [13, 22]. We attributed our finding primarily to misregistration artifacts caused by misalignment due to through-plane motion artifacts in the acquired lower-*b*-value images. Advanced co-registration software could potentially increase image quality as proposed by Agkagi et al [11].

SNR significantly dropped in cDWI compared to *b* = 600 s/mm^2^ images. Likewise, a study by Tamura et al reported decreased SNR in cDWI images in breast cancer patients [23]. In a study by Gatidis et al, background intensity variation of cDWI increased monotonically with increasing *b* values [24]. As the *b* values increase, signal intensity decreases, thus limiting the maximum *b* value applicable for cDWI [25].

Comparing qualitative image parameters between computed and directly acquired images revealed non-inferiority in cDWI images. Furthermore, aSNR, aCNR, and CR were significantly higher in cDWI images, which can be explained by the reduced acquisition time. These findings seem to be particularly interesting in light of the markedly reduced acquisition time for high-resolution cDWI compared to directly acquired high-resolution DWI in our cohort. However, it must be borne in mind that the rFOV-DWI sequence was employed as a study protocol. A scan time of 15 min as employed herein is not applicable to clinical routine. Hence, cDWI enables the generation of high-*b*-value images at a clinically justifiable examination time, supporting recent studies on the implementation of abbreviated MRI protocols in IPMNs [26].

Our study has limitations. First, cDWI images were primarily compared to standard DWI images at *b* = 600 s/mm^2^ as routinely performed at our institution. However, we are well aware that other institutions might use different *b*-values for pancreatic DWI. Hence, additional studies should elucidate if the potential benefits as shown in our study hold true for different *b* values. Second, we focused on a mono-exponential diffusion model. Other models, e.g., bi- tri-exponential diffusion models, could further add diagnostic value. Third, EUS was chosen as the standard of reference for most of our cases. However, as this is an operator-depended modality, a potential bias could not finally be ruled out.

Fourth, due to the above-mentioned limitations and particularly its retrospective and single-center nature, further prospective and multicenter cohorts need to ascertain the herein**-**found results. Fifth, we excluded all patients with IPMNs < 10 mm. However, patients with IPMNs > 10 mm were by far the most often referred group. Moreover, in our experience and also in view of previously published studies, the risk of malignant transformation/presence of solid nodules in cysts < 10 mm is extremely low [27, 28].

## Conclusion

Our study underlines the potential of high-*b*-value cDWI combined with high-resolution rFOV-DWI, in the imaging of pancreatic IPMN. These images provide better identification of solid nodules with no time penalty. Improvement of MRI might be important with the development of surveillance.

### Supplementary Information

Below is the link to the electronic supplementary material.

Supplementary file1 (PDF 65 KB)

## Abbreviations

aCNR: Apparent contrast-to-noise ratio
aSNR: Apparent signal-to-noise ratio
BD-IPMN: Branch duct IPMN
cDWI: Computed diffusion-weighted imaging
CR: Contrast ratio
fFOV: Full field of view
MT-IPMN: Mixed-type IPMN
rFOV: Reduced field of view
SI: Signal intensity
ssEPI: Single-shot echo-planar imaging

## Declarations

### Guarantor

The scientific guarantor of this publication is Rickmer Braren.

### Statistics and biometry

No complex statistical methods were necessary for this paper.

### Methodology

• retrospective

• observational

• performed at one institution

## References

[1] Klibansky DA, Reid-Lombardo KM, Gordon SR, Gardner TB (2012). The clinical relevance of the increasing incidence of intraductal papillary mucinous neoplasm. Clin Gastroenterol Hepatol.

[2] Adsay V, Mino-Kenudson M, Furukawa T (2016). Pathologic evaluation and reporting of intraductal papillary mucinous neoplasms of the pancreas and other tumoral intraepithelial neoplasms of pancreatobiliary tract: recommendations of Verona Consensus Meeting. Ann Surg.

[3] Tanaka M, Fernández-Del Castillo C, Kamisawa T (2017). Revisions of international consensus Fukuoka guidelines for the management of IPMN of the pancreas. Pancreatology.

[4] European Study Group on Cystic Tumours of the Pancreas (2018). European evidence-based guidelines on pancreatic cystic neoplasms. Gut.

[5] Vege SS, Ziring B, Jain R, Moayyedi P (2015) American gastroenterological association institute guideline on the diagnosis and management of asymptomatic neoplastic pancreatic cysts. Gastroenterology 148:819–822 quize812-813 10.1053/j.gastro.2015.01.015 25805375

[6] Kim KW, Park SH, Pyo J (2014). Imaging features to distinguish malignant and benign branch-duct type intraductal papillary mucinous neoplasms of the pancreas: a meta-analysis. Ann Surg.

[7] Bammer R (2003). Basic principles of diffusion-weighted imaging. Eur J Radiol.

[8] Jang KM, Kim SH, Min JH (2014). Value of diffusion-weighted MRI for differentiating malignant from benign intraductal papillary mucinous neoplasms of the pancreas. AJR Am J Roentgenol.

[9] Fukukura Y, Kumagae Y, Hakamada H (2017). Computed diffusion-weighted MR imaging for visualization of pancreatic adenocarcinoma: comparison with acquired diffusion-weighted imaging. Eur J Radiol.

[10] Dietrich O, Biffar A, Baur-Melnyk A, Reiser MF (2010). Technical aspects of MR diffusion imaging of the body. Eur J Radiol.

[11] Akagi M, Nakamura Y, Higaki T (2018). Preliminary results of high-precision computed diffusion weighted imaging for the diagnosis of hepatocellular carcinoma at 3 Tesla. J Comput Assist Tomogr.

[12] Blackledge MD, Leach MO, Collins DJ, Koh DM (2011). Computed diffusion-weighted MR imaging may improve tumor detection. Radiology.

[13] Ichikawa S, Kromrey ML, Motosugi U, Onishi H (2021). Optimal target b-value on computed diffusion-weighted magnetic resonance imaging for visualization of pancreatic ductal adenocarcinoma and focal autoimmune pancreatitis. Abdom Radiol (NY).

[14] Rosenkrantz AB, Chandarana H, Hindman N (2013). Computed diffusion-weighted imaging of the prostate at 3 T: impact on image quality and tumour detection. Eur Radiol.

[15] Kim H, Lee JM, Yoon JH (2015). Reduced field-of-view diffusion-weighted magnetic resonance imaging of the pancreas: comparison with conventional single-shot echo-planar imaging. Korean J Radiol.

[16] Harder FN, Kamal O, Kaissis GA (2020). Qualitative and quantitative comparison of respiratory triggered reduced field-of-view (FOV) versus full FOV diffusion weighted imaging (DWI) in pancreatic pathologies. Acad Radiol.

[17] Harder FN, Jung E, McTavish S (2022). High-resolution, high b-value computed diffusion-weighted imaging improves detection of pancreatic ductal adenocarcinoma. Cancers (Basel).

[18] Xu F, Liang Y, Guo W (2021). Diagnostic performance of diffusion-weighted imaging for differentiating malignant from benign intraductal papillary mucinous neoplasms of the pancreas: a systematic review and meta-analysis. Front Oncol.

[19] Choi SY, Kim JH, Yu MH, Eun HW, Lee HK, Han JK (2017). Diagnostic performance and imaging features for predicting the malignant potential of intraductal papillary mucinous neoplasm of the pancreas: a comparison of EUS, contrast-enhanced CT and MRI. Abdom Radiol (NY).

[20] Hwang J, Kim YK, Min JH, Jeong WK, Hong SS, Kim HJ (2018). Comparison between MRI with MR cholangiopancreatography and endoscopic ultrasonography for differentiating malignant from benign mucinous neoplasms of the pancreas. Eur Radiol.

[21] Kim YC, Choi JY, Chung YE (2010). Comparison of MRI and endoscopic ultrasound in the characterization of pancreatic cystic lesions. AJR Am J Roentgenol.

[22] Tokunaga K, Arizono S, Shimizu H (2020). Optimizing b-values for accurate depiction of pancreatic cancer with tumor-associated pancreatitis on computed diffusion-weighted imaging. Clin Imaging.

[23] Tamura T, Takasu M, Higaki T (2019). How to improve the conspicuity of breast tumors on computed high b-value diffusion-weighted imaging. Magn Reson Med Sci.

[24] Gatidis S, Schmidt H, Martirosian P, Nikolaou K, Schwenzer NF (2016). Apparent diffusion coefficient-dependent voxelwise computed diffusion-weighted imaging: an approach for improving SNR and reducing T2 shine-through effects. J Magn Reson Imaging.

[25] Higaki T, Nakamura Y, Tatsugami F (2018). Introduction to the technical aspects of computed diffusion-weighted imaging for radiologists. Radiographics.

[26] Delaney FT, Fenlon HM, Cronin CG (2021). An abbreviated MRI protocol for surveillance of cystic pancreatic lesions. Abdom Radiol (NY).

[27] Kromrey ML, Bülow R, Hübner J (2018). Prospective study on the incidence, prevalence and 5-year pancreatic-related mortality of pancreatic cysts in a population-based study. Gut.

[28] Capurso G, Crippa S, Vanella G (2020). Factors associated with the risk of progression of low-risk branch-duct intraductal papillary mucinous neoplasms. JAMA Netw Open.

