# Supplementary figures and images for: Computed high-b-value high-resolution DWI improves solid lesion detection in IPMN of the pancreas
Source: Eur Radiol. 2023 May 3;33(10):6892–901. doi: 10.1007/s00330-023-09661-6 (PMC10511579; doi:10.1007/s00330-023-09661-6)

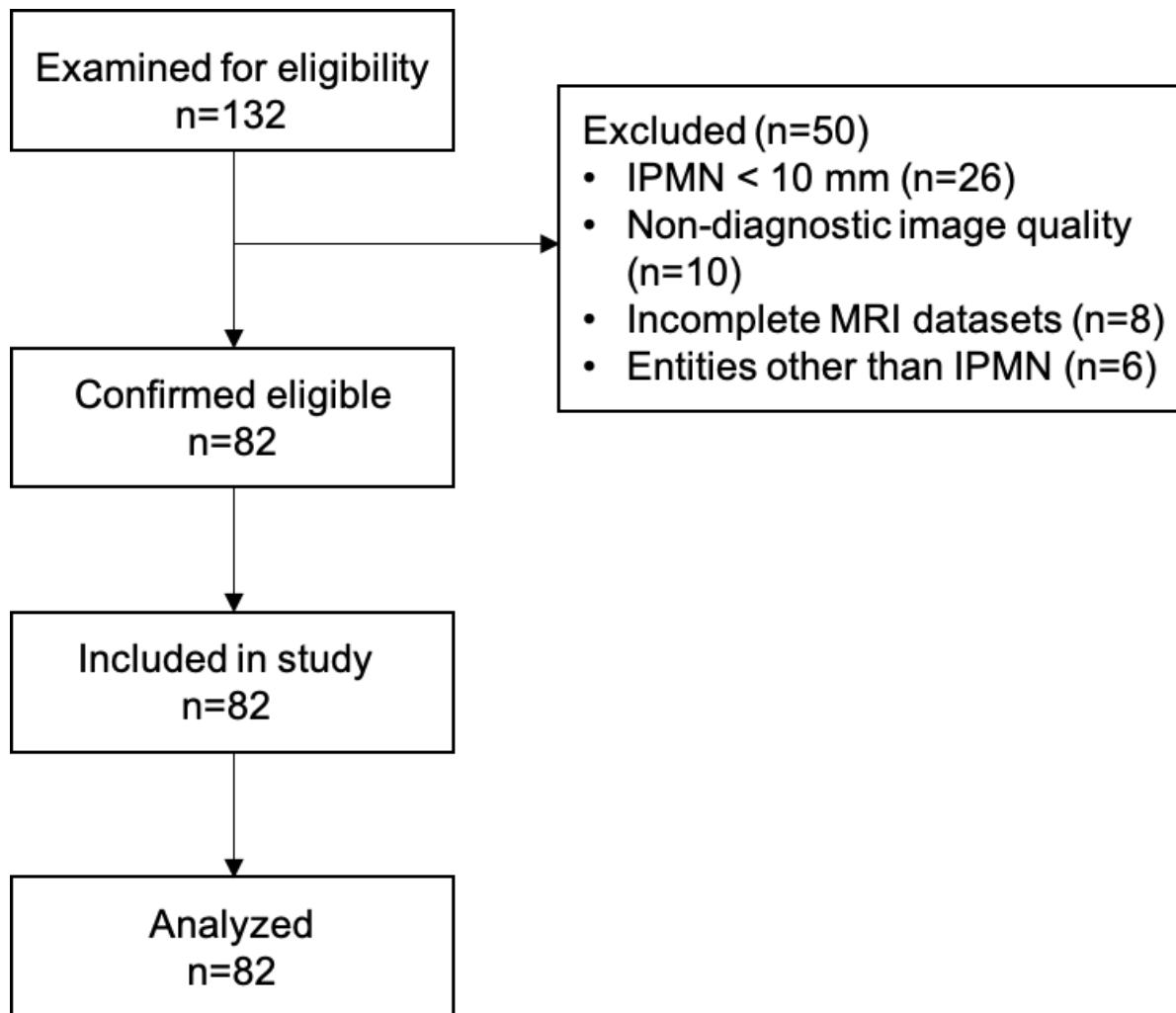

S1. Patient inclusion flowchart

Supplement: Supplementary file 1 — Supplementary file1 (PDF 65 KB) [file 330_2023_9661_MOESM1_ESM.pdf]
